# Mitral Annular Disjunction and “Pseudo” Mitral Annular Disjunction in Mitral Valve Prolapse

**DOI:** 10.1016/j.jaccas.2024.102436

**Published:** 2024-08-21

**Authors:** Tam T. Doan, Shaine A. Morris

**Affiliations:** a Texas Children's Hospital, Houston, Texas, USA; b Baylor College of Medicine, Houston, Texas, USA

We are intrigued by the cases reported by Alfares et al^1^ and commend the authors for highlighting the association between mitral annular disjunction (MAD) and sudden cardiac arrest in pediatric patients. However, we would like to note that their measurement of MAD distance included both MAD and “pseudo” MAD due to mitral valve prolapse (MVP), leading to an overestimation of MAD distance.^2^

**Figure 1:**
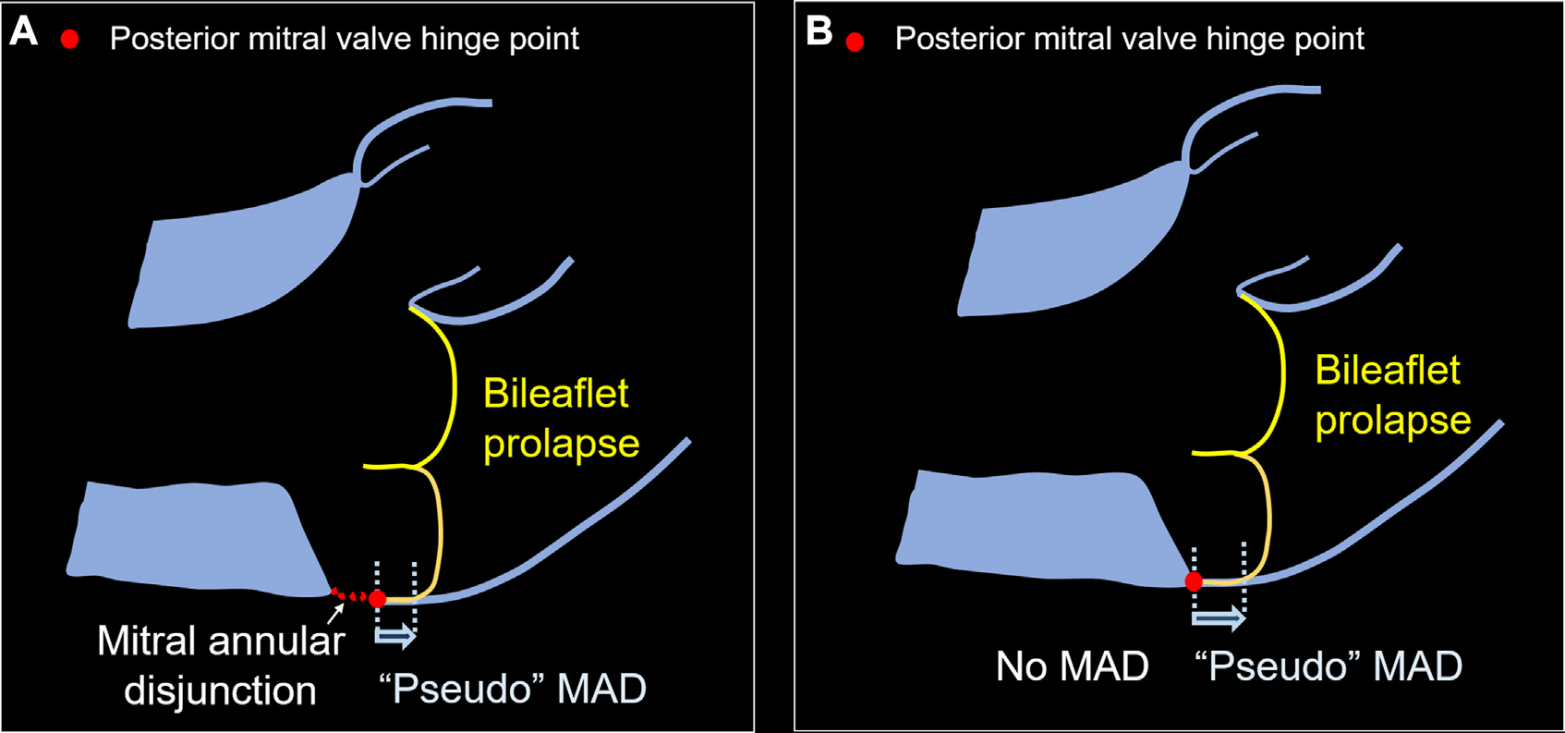
Diagram of MVP With and Without MAD The red dot depicted the posterior mitral valve hinge point. (A) MAD and “pseudo” MAD and (B) “pseudo” MAD without MAD. MAD = mitral annular disjunction; MVP = mitral valve prolapse.

MAD refers to an anatomical separation between the posterior mitral valve annulus and the left ventricular myocardium.^2^^,^^3^ Hutchins et al^3^ conducted a review of 900 postmortem hearts, revealing MAD in most specimens with MVP (23 of 25) and 42 with MAD only. MAD can be identified on an echocardiogram’s parasternal long-axis view and long-axis cine of the left ventricular outflow on cardiac magnetic resonance imaging.^2^ We developed a reproducible method for identifying MAD on echocardiograms in 185 pediatric patients with Marfan syndrome. MAD was present in most patients with MVP and was present without MVP in 32% of 185 patients.^4^ We also demonstrated a 96% agreement between echocardiography and CMR in detecting MAD (kappa = 0.89; *P* < 0.0001).^4^ Assessing MAD necessitated a meticulous image review to accurately track the posterior mitral valve hinge point, especially in MVP cases, to recognize “pseudo” MAD and prevent overestimation of MAD distance (Figure 1).^4^

It has been proposed that MAD distance could serve as an indicator of MAD severity and potentially act as a biomarker of clinical outcomes. Therefore, it is important to establish a reliable method for measuring MAD, and distinguishing MAD and “pseudo” MAD in patients with MVP may be important.
